# Lung tissue expression of epithelial injury markers is associated with acute lung injury severity but does not discriminate sepsis from ARDS

**DOI:** 10.1186/s12931-024-02761-x

**Published:** 2024-03-18

**Authors:** Natália de Souza Xavier Costa, Giovana da Costa Sigrist, Alexandre Santos Schalch, Luciano Belotti, Marisa Dolhnikoff, Luiz Fernando Ferraz da Silva

**Affiliations:** 1 Departamento de Patologia, Faculdade de Medicina da Universidade de São Paulo, São Paulo, LIM-05 Brazil; 2 Serviço de Verificação de Óbitos da Capital, Universidade de São Paulo, São Paulo, Brazil

**Keywords:** ARDS, Sepsis, Receptor for Advanced Glycation endproducts, Elafin, Surfactant protein D

## Abstract

**Background:**

Acute respiratory distress syndrome (ARDS) is a common cause of respiratory failure in critically ill patients, and diffuse alveolar damage (DAD) is considered its histological hallmark. Sepsis is one of the most common aetiology of ARDS with the highest case-fatality rate. Identifying ARDS patients and differentiate them from other causes of acute respiratory failure remains a challenge. To address this, many studies have focused on identifying biomarkers that can help assess lung epithelial injury. However, there is scarce information available regarding the tissue expression of these markers. Evaluating the expression of elafin, RAGE, and SP-D in lung tissue offers a potential bridge between serological markers and the underlying histopathological changes. Therefore, we hypothesize that the expression of epithelial injury markers varies between sepsis and ARDS as well as according to its severity.

**Methods:**

We compared the post-mortem lung tissue expression of the epithelial injury markers RAGE, SP-D, and elafin of patients that died of sepsis, ARDS, and controls that died from non-pulmonary causes. Lung tissue was collected during routine autopsy and protein expression was assessed by immunohistochemistry. We also assessed the lung injury by a semi-quantitative analysis.

**Results:**

We observed that all features of DAD were milder in septic group compared to ARDS group. Elafin tissue expression was increased and SP-D was decreased in the sepsis and ARDS groups. Severe ARDS expressed higher levels of elafin and RAGE, and they were negatively correlated with PaO_2_/FiO_2_ ratio, and positively correlated with bronchopneumonia percentage and hyaline membrane score. RAGE tissue expression was negatively correlated with mechanical ventilation duration in both ARDS and septic groups. In septic patients, elafin was positively correlated with ICU admission length, SP-D was positively correlated with serum lactate and RAGE was correlated with C-reactive protein.

**Conclusions:**

Lung tissue expression of elafin and RAGE, but not SP-D, is associated with ARDS severity, but does not discriminate sepsis patients from ARDS patients.

**Supplementary Information:**

The online version contains supplementary material available at 10.1186/s12931-024-02761-x.

## Introduction

Acute respiratory distress syndrome (ARDS) is a common cause of respiratory failure in critically ill patients and is defined by acute onset of noncardiogenic pulmonary oedema and hypoxaemia that requires mechanical ventilation [1]. ARDS is an alarming complication that usually develops in patients with conditions that may induce systemic inflammation, such as sepsis, pneumonia, and major trauma [2]. Sepsis is one of the most common aetiology of ARDS and these patients present the highest case-fatality rate [3].

Histologically, diffuse alveolar damage (DAD) is considered the hallmark of the acute phase of ARDS and it is characterized by an initial exudative phase with oedema, hyaline membrane formation, and interstitial acute inflammation, followed by a fibroproliferative phase with loose organizing fibrosis mostly within the alveolar septa, and type II pneumocyte hyperplasia [4]. However, clinical and autopsy studies suggest that only one-half of patients who meet the clinical definition of ARDS have DAD [5, 6]. Most importantly, the subgroup of patients with ARDS who also have DAD appears to have increased mortality [5].

ARDS diagnosis relays only on clinical-radiological variables and features relating to histology are not included in the definition because evaluating these variables is invasive and considered clinically unfeasible [4]. More recently, a growing body of evidence shows that a more feasible way of assessing lung epithelial injury could be through specific markers in plasma, such as the surfactant protein D (SP-D) and the receptor for advanced glycation endproducts (RAGE). Elafin, another potential marker, is described as a potent protease inhibitor. During inflammatory processes in the lungs, the excessive protease activity may lead to damage to the alveolar epithelial–capillary endothelial barrier resulting in the production of pulmonary exudative oedema within the alveolar space [7].

Furthermore, lung tissue information about DAD and about the disruption of the alveolar epithelial–capillary barrier is relevant since patients with DAD are about five times as likely to die of refractory hypoxemia than patients without DAD [8]. Despite the critical importance of epithelial injury markers, only limited studies have explored their expression in the lung tissue of ARDS and septic patients. Evaluating the expression of elafin, RAGE, and SP-D in lung tissue offers a potential bridge between serological markers and the underlying histopathological changes. We hypothesize that the expression of epithelial injury markers varies between sepsis and ARDS as well as according to its severity. To evaluate this hypothesis this study compared the tissue expression of elafin, RAGE, and SP-D in patients who died of sepsis, ARDS (and its severity), and controls with non-pulmonary causes of death. Additionally, we correlated these findings with relevant clinical variables. Through a comprehensive characterization of the lung injury assessed by semi-quantitative histological scores in ARDS and sepsis patients, we further provided a more nuanced understanding of the association between epithelial injury markers, histological changes, and clinical variables.

## Materials and methods

### Study population

This retrospective project was approved by the review board for the human ethics committee of Sao Paulo University (CAPPesq-FMUSP; CAAE: 67771417.0.0000.0068).

We have selected 47 patients with a clinical diagnosis of ARDS as defined by the Berlin definition [9], in addition to histological findings of DAD. We further selected 30 patients with clinical diagnosis of sepsis defined according to Singer et al. [10] and without clinical criteria for ARDS. For both groups, we excluded individuals with a previous history of smoking and/or chronic lung disease and individuals that the medical records were not available and that laboratory exams necessary for the diagnosis (e.g., serum lactate, cultures, arterial blood gas analysis, and lung image exams) were not available or not performed.

As controls, we selected 27 patients who died from non-pulmonary causes, without a previous history of smoking and/or chronic lung disease and/or mechanical ventilation, with preserved lung tissue at histological analysis.

All patients had their lung tissue collected during the routine autopsy performed at the Sao Paulo Autopsy Service – University of Sao Paulo (SVOC-USP) between 2002 and 2014. To better represent the lung tissue, we have selected two to three post-mortem lung samples of each case, avoiding areas of abscess and/or necrosis and/or additional relevant tissue destruction which would compromise all the immunostainings and analysis as blocks with limited amount of tissue.

### Clinical data

Clinical data were retrospectively collected from the medical records during the hospital admission period and laboratory exams performed 24 h prior to death. Since the control individuals died mostly from sudden deaths, we did not had access to their laboratory exams.

We collected the following clinical information: duration of hospitalization, mechanical ventilation (MV), ICU admission length, Sequential Organ Failure Assessment (SOFA) score, and information regarding mechanical ventilation, such as the fraction of inspired oxygen (FiO_2_), positive end-expiratory pressure (PEEP), partial pressure of arterial oxygen (PaO_2_) to FiO_2_ ratio. We also collected information regarding the arterial blood gas analysis such as arterial blood pH, PaO_2_, partial pressure of carbon dioxide (PaCO_2_), bicarbonate (HCO_3_), base excess, oxygen saturation (SO_2_), the fraction of oxyhaemoglobin (FO_2_Hb), Fraction of carboxyhaemoglobin (FCO_2_Hb), Fraction of methaemoglobin (FMetHb), Fraction of deoxyhaemoglobin (FHHb), and the oxygen tension at which haemoglobin is 50% saturated (p50).

We collected information about the full blood count in addition to serum lactate and C-reactive protein (CRP).

### Semi-quantitative histological assessment

Slides stained with haematoxylin and eosin (H&E) were blinded and evaluated by an experienced pathologist who quantified the proportion (%) of the following histological patterns: normal lung tissue, exudative DAD, fibroproliferative DAD, and acute bronchopneumonia. The histological criteria used were: (a) normal tissue: lung parenchyma with normal histology or minimal non-specific changes as mild oedema and congestion; (b) exudative DAD: interstitial and/or intra-alveolar oedema, interstitial inflammation, variable amounts of alveolar haemorrhage and fibrin deposition, intra-alveolar hyaline membranes and type II pneumocyte hyperplasia; (c) fibroproliferative DAD: any degree of fibroblastic proliferation within the interstitium and/or alveolar spaces, including loose aggregates of fibroblasts admixed with scattered inflammatory cells and collagen deposition, intermingled with areas with hyaline membranes or densely fibrotic areas [11]. To further explore the features of each DAD pattern, each slide was scored for septal thickening, oedema, inflammation, hyaline membrane, alveolar haemorrhage, and proliferation of type II pneumocytes, with the following graduation: 0- absent, 1-mild, 2-moderate, and 3- severe. The cases were also classified by the type of inflammation: 0- Absent, 1- Predominantly neutrophilic inflammation, 2- Predominantly lymphomononuclear inflammation, and 3- Mixed inflammation [12, 13].

### Immunohistochemistry

Lung tissue was immunostained using anti-elafin (Abcam, UK; cat. #ab81681; 1:300), anti-RAGE (Santa Cruz Biotechnology, Dallas, TX, USA; cat. #sc-365,154; 1:300), and anti-SP-D (Santa Cruz Biotechnology, Dallas, TX, USA; cat. #sc-25,324; 1:750). We also stained the lung slides with Sirius Red for collagen quantification. The epithelial damage markers and collagen were quantified in the lung septa, excluding airways, large blood vessels (only capillaries were not excluded), pleura, and loose cells in the alveolar space [14]. Positive-stained area per septa length (µm²/µm) was measure measured in 20 high-power fields using the Image-Pro Plus 4.1 software (Media Cybernetics, Silver Spring, MD, USA).

### Statistical analysis

SPSS 23 software (SPSS Inc/IBM Chicago, USA) was used for the statistical analyses. GraphPad Prism 7 (GraphPad Software, La Jolla, CA, USA) and RStudio, version 4.1.1 (RStudio, PBC, Boston, MA, USA) were used for data visualization.

Categorical variables were analysed using the chi-square test and simple correspondence analysis (ANACOR). We further analysed the dependency relationships between each pair of categories using the adjusted standardized residuals of the chi-square test, adopting an alpha value of 0.05.

Quantitative variables distribution was assessed by the Shapiro-Wilk normality test. Depending on the data distribution, T-student or Mann-Whitney tests were used to compare two groups and Kruskal–Wallis or one-way ANOVA tests, followed by the Bonferroni or Tukey posthoc test to compare four groups. Statistical difference was assumed at the 5% significance level. The coefficient of variation (CV) was calculated for every case by dividing the standard deviation by the mean. We performed the Spearman correlation test between variables; coefficients (r) were considered statistically significant at *p* < 0.05.

As a strategy of data analysis, we compared all histological variables among the 3 groups: Control, Sepsis, and ARDS. We also compared the sepsis group to the subgroups formed by the division of the ARDS group according to its severity: Mild: 200 mmHg < PaO_2_/FiO_2_ ratio ≤ 300 mmHg, Moderate: 100 mmHg < PaO_2_/FiO_2_ ratio ≤ 200 mmHg, and Severe: PaO_2_/FiO_2_ ratio ≤ 100 mmHg. Within the ARDS group, we compared the pulmonary ARDS and extrapulmonary ARDS. Data from the laboratory exams were compared between the ARDS and sepsis groups and correlated with histological variables.

## Results

### Demographics and clinical features

The demographics and main comorbidities are presented in Table 1.

**Table 1:** Demographic and clinical characteristics of Control, Septic and ARDS patients

		Control (*n* = 27)	Sepsis (*n* = 30)	ARDS (*n* = 47)	*p*-value
**Age in years**, median (IQR)		62 (20)	59 (31)	55 (22)	0.079
**BMI (Kg/m**^**2**^**)**, median (IQR)		24.1 (3.68)	25.7 (6.68)	24.3 (6.03)	0.310
**Sex**, n (%)					
	Male	12 (44.4%)	10 (33.3%)	24 (51.1%)	0.311
	Female	15 (55.6%)	20 (66.7%)	23 (48.9%)	
**Self-declared Race**, n (%)					
	White	20 (74.1%)	24 (80%)	32 (68.1%)	0.512
	Afro-descendent	7 (25.9%)	6 (20%)	15 (31.9%)	
**Comorbidities**, n (%)					
	SAH	20 (74.1%)	19 (63.3%)	20 (42.6%)	**0.021**
	Cardiopathy	12 (46.2%)	8 (26.7%)	5 (10.6%)	**0.003**
	Vascular disease	15 (57.7%)	5 (16.7%)	5 (10.6%)	**< 0.0001**
	Diabetes mellitus	10 (37%)	11 (36.7%)	11 (23.4%)	0.335
	Obesity	4 (14.8%)	7 (25.9%)	4 (10.3%)	0.229
	Hepatic disease	4 (15.4%)	8 (26.7%)	9 (19.1%)	0.556
	Neoplasia	2 (7.4%)	5 (16.7%)	14 (29.8%)	0.059
	Alcoholism	2 (7.4%)	3 (10%)	6 (12.8%)	0.770
	Chronic renal disease	0	4 (13.3%)	6 (15.8%)	0.056
	Immunosuppression	0	3 (10%)	14 (29.8%)	**0.041**
	Nervous system disease	0	3 (10%)	6 (15.8%)	0.173
	Pulmonary Hypertension	0	3 (10%)	2 (4.3%)	0.251
	HIV +	0	1 (3.3%)	3 (6.4%)	0.383
**Period of hospitalization in days**, median (IQR)		-	7.5 (24)	13 (14)	0.101
**Mechanical ventilation duration in days**, median (IQR)		-	1 (2)	3 (7)	**< 0.0001**
**Period of ICU stay in days**, median (IQR)		-	3.5 (7)	5 (10)	0.154
**SOFA score**, median (IQR)		-	15 (6)	15 (4)	0.961
**Lungs weight in grams**, median (IQR)		-	1026 (456)	1575 (756)	**< 0.0001**
**Mechanical Ventilation**, n (%)		-	25 (83.3%)	47 (100%)	**0.031**
FiO_2_ (%), median (IQR)		-	100 (50)*	90 (50)	0.895
PEEP (cmH_2_O), median (IQR)		-	8 (5)**	10 (6)	**< 0.0001**
PaO_2_/FiO_2_ ratio, median (IQR)		-	120.5 (136)***	95 (124)	0.218

BMI, Body Mass Index; FiO2, Inspired Fraction of Oxygen HIV, Human Immunodeficiency Virus; ICU, Intensive Care Unit; IQR, Interquartile range; PaO2, partial pressure of arterial oxygen; PEEP, positive end-expiratory pressure; SAH, Systemic Arterial Hypertension; SOFA, Sequential Organ Failure Assessment. All the variables have the number of individuals (n) in each group as indicated in the top of the table, except for: * Sepsis group: *n* = 20; **Sepsis group: *n* = 21; and ***Sepsis group: *n* = 15

Control individuals (12 Males /15 Females) had a median age of 62 years (range 27–93). Most of them died of sudden deaths, such as acute myocardial infarction (37%, *n* = 10), heart insufficiency (37%, *n* = 10), hypovolemic shock (11.1%, *n* = 3), or stroke (3.7%, *n* = 1). The other cases died from cancer (7.4% *n* = 2) and acute myocarditis (3.7%, *n* = 1).

Sepsis cases (10 Males, 20 Females) had a median age of 59 years (range 27–87), with pulmonary (*n* = 5) or extra-pulmonary (*n* = 25) focus of infection. Twenty-five (83.3%) cases required mechanical ventilation. ARDS group (24 Males, 23 Females) had a median age of 55 years (range 19–88) and included patients with pulmonary (*n* = 25) and non-pulmonary ARDS (*n* = 22).

We did not observe a statistical difference in the period of hospitalization, ICU stay, and SOFA score between the sepsis and ARDS groups. While not all cases of sepsis required MV (*n* = 25 out of 30), all patients in the ARDS group did, with the ARDS group exhibiting a significantly longer duration of MV (*p* < 0.0001).

We did not observe statistical difference in the FiO_2_ (sepsis group: *n* = 20; ARDS group: *n* = 47), and PaO_2_/FiO_2_ ratio (sepsis group: *n* = 15; ARDS group: *n* = 47) between the sepsis and ARDS groups. Nevertheless, the ARDS group exhibited higher lung weight (*p* < 0.0001), and PEEP (sepsis group: *n* = 21; ARDS group: *n* = 47; *p* < 0.0001) compared to sepsis (Table 1). The most common associated conditions with the respiratory failure or sepsis are presented in Table 2.

**Table 2:** Relevant associated conditions of the Sepsis and ARDS groups

Relevant Associated Conditions*, n (%)		Sepsis (*n* = 30)	ARDS (*n* = 47)
Extrapulmonary Infection		27 (90%)	20 (42.6%)
Bronchopneumonia		5 (16.7%)	25 (53.2%)
	Bacterial	5 (16.7%)	14 (29.8%)
	Influenza H1N1	0	6 (12.8%)
	Pneumocystosis**	0	2 (4.3%)
	Cytomegalovirus	0	2 (4.3%)
	Respiratory syncytial virus	0	1 (2.1%)
	Aspergillosis	0	1 (2.1%)
Acute renal failure		10 (33.3%)	26 (55.3%)
Cardiovascular diseases		7 (23.3%)	11 (23.4%)
Liver diseases		7 (23.3%)	9 (19.1%)
Neurologic diseases		4 (13.3%)	4 (8.5%)
Gastrointestinal bleeding		3 (10%)	4 (8.5%)
Pulmonary thromboembolism		2 (6.7%)	4 (8.5%)

*Some patients may have more than one associated condition. **One case tested positive for *Pneumocystis jiroveci* and for Cytomegalovirus in bronchoalveolar lavage

All the laboratory tests from the sepsis and ARDS groups are presented in Table S1 (Additional Table 1).

### Histological features and DAD assessment

**Fig. 1:**
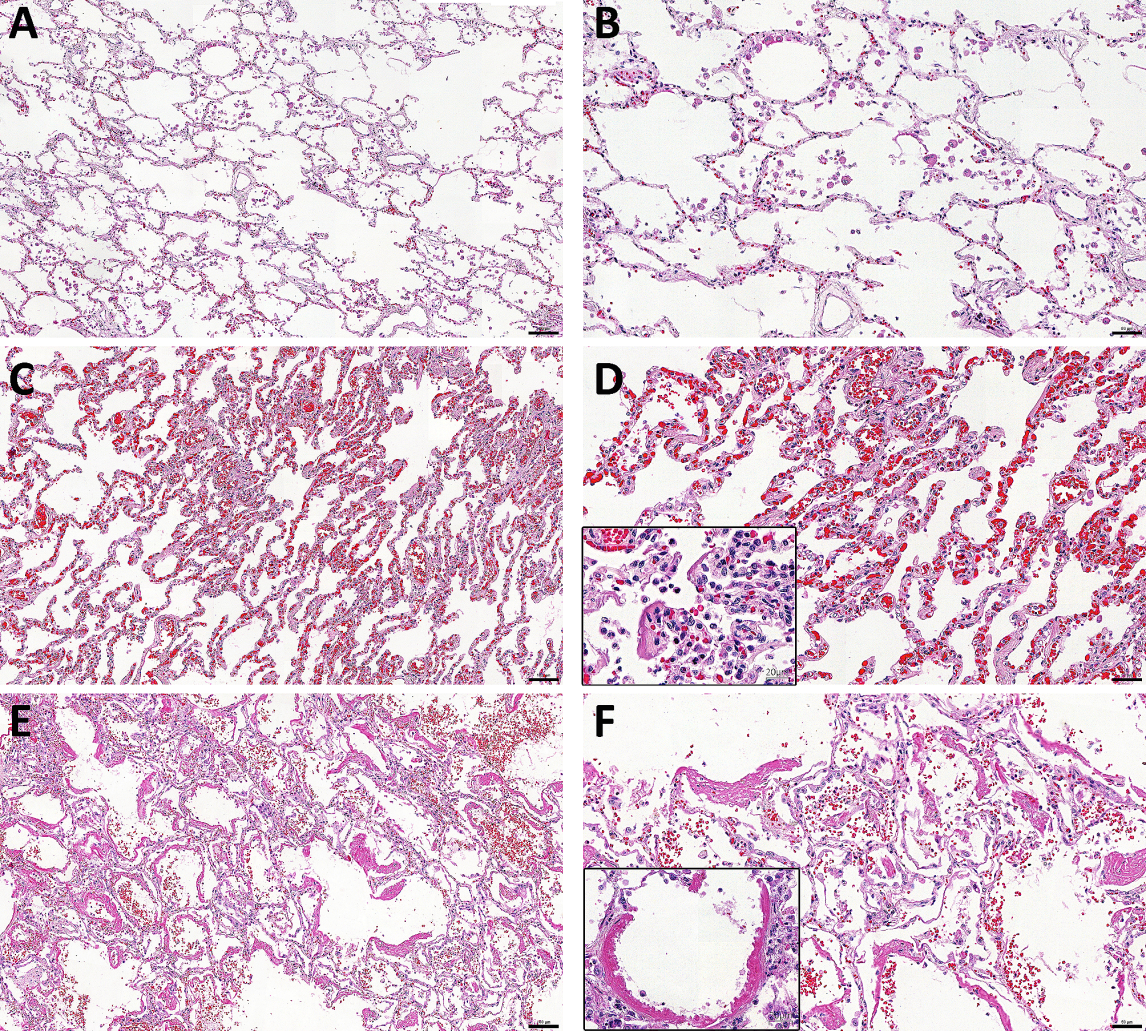
Representative H&E-stained Photomicrographs of Control, Sepsis and ARDS lung tissue. **A** (10x) and **B** (20x) - Control group: Preserved alveolar parenchyma. Since most of this group have heart conditions related cause of death, it is possible to observe a moderate presence of macrophages in the lung tissue. **C** (10x) and D (20x) – Sepsis Group: Intense congestion and mild inflammatory infiltrate. In detail (40x), a focal area of hyaline membrane formation is shown. **E** (10x) and F (20x) – ARDS group: Intense hyaline membrane formation and mild alveolar haemorrhage. In detail (40x), hyaline membrane formation upon a denuded alveolar basement membrane. Scale bar 10x = 100 μm. Scale bar 20x = 50 μm. Scale bar 40x = 20 μm

Figure 1 shows representative microphotographs of H&E-stained slides of each group. As expected, all lung tissue injury features assessed in the histological analysis showed a statistical difference between the groups ARDS and sepsis and the control group (Table 3). We further observed that ARDS group showed higher bronchopneumonia percentage (*p* = 0.025) and higher scores for inflammation (*p* = 0.015), hyaline membrane (*p* = 0.001), haemorrhage (*p* = 0.019), and septal thickening (*p* = 0.049) than the sepsis group (Table 3).

**Table 3:** Semi-quantitative histological analysis

				ARDS			
	Control (*n* = 27)	Sepsis (*n* = 30)		All cases (*n* = 47)	Mild (*n* = 8)	Moderate (*n* = 14)	Severe (*n* = 25)
**Histological Patterns (%)**, median (range)							
Normal tissue	100 (95–100)	10 (0–55)^A^		2.5 (0–50)^A^	10 (0–50)	0 (0–50)^B^	2.5 (0–40)^B^
Exudative DAD	0	57.5 (10–100)^A^		60 (28–100)^A^	55 (35–93)	60 (28–100)	60 (30–100)
Fibroproliferative DAD	0	0 (0–40)^A^		0 (0–45)^A^	2.5 (0–10)	2.5 (0–45)	0 (0–45)
Bronchopneumonia	0^B^	0 (0–20)		0 (0–48)^B^	0 (0–25)	0 (0–40)	0 (0–48)
**Scores**, median (range)							
Septal thickening	0 (0–1)	1 (0–3)^A^		2 (1–3)^AB^	1 (0–3)	1.5 (1–2)^B^	2 (1–3)^B^
Oedema	0	1.5 (1–3)^A^		2 (0–3)^A^	1 (1–3)	2 (0–3)	2 (1–3)^B^
Inflammation	0 (0–1)	1 (1–2)^A^		2 (1–3)^AB^	2 (1–2)	2 (1–3)^B^	2 (1–3)^B^
Hyaline Membrane	0	1 (0–2)^A^		1 (1–3)^AB^	1 (1–3)	1 (1–3)^B^	2 (1–3)^B^
Alveolar Haemorrhage	0 (0–1)	1 (0–3)^A^		1 (0–3)^AB^	1 (0–3)	1 (0–3)	1 (0–3)
Proliferation of type II pneumocytes	0	1 (0–2)^A^		1 (0–3)^A^	1.5 (0–3)	2 (1–3)	1 (1–3)
**Inflammation pattern**, n (%)							
Absent	23 (85.5%)	0		0	0	0	0
Neutrophilic	0	5 (16.7%)		6 (12.8%)	1 (12.5%)	2 (14.3%)	3 (12%)
Lymphomononuclear	4 (14.8%)	11 (36.7%)		11 (23.4%)	2 (25%)	3 (21.4%)	6 (24%)
Mixed	0	14 (46.7%)		30 (63.8%)	5 (62.5%)	9 (64.3%)	16 (64%)

^A^ *p* < 0.05 compared to the control group. ^B^ *p* < 0.05 compared to the sepsis group. Inflammation Pattern: χ² = 89.218; *p* < 0.0001

Regarding the inflammation pattern, the ARDS group had 12.8% of the cases predominantly neutrophilic, 23.4% of the cases predominantly lymphomononuclear, and 63.8% of the cases presenting mixed inflammation. The sepsis group had 16.7% of the cases predominantly neutrophilic, 36.7% of the cases predominantly lymphomononuclear, and 46.7% of the cases presenting mixed inflammation. ANACOR biplots visually show the differences among the groups in the histological scores (Figures S1-S7, Additional file 1).

When we compared the ARDS subgroups according to their severity to the sepsis group, we observed that normal tissue percentage was lower in the moderate ARDS (*p* = 0.032) and severe ARDS (*p* = 0.045) compared to the sepsis group. Inflammation and hyaline membrane scores were higher in the moderate ARDS (*p* = 0.005 and *p* < 0.0001, respectively) and severe ARDS (*p* = 0.017 and *p* = 0.031, respectively) than in the sepsis group. Septal thickening and oedema scores were also higher in the severe ARDS compared to the sepsis (*p* = 0.038 and *p* = 0.006, respectively).

In addition, within the ARDS group, we only observed higher oedema score in the pulmonary ARDS (*p* = 0.020) (Figure S8, Additional file 1).

### Immunohistochemical assessment of the epithelial injury markers elafin, RAGE and SP-D

**Fig. 2:**
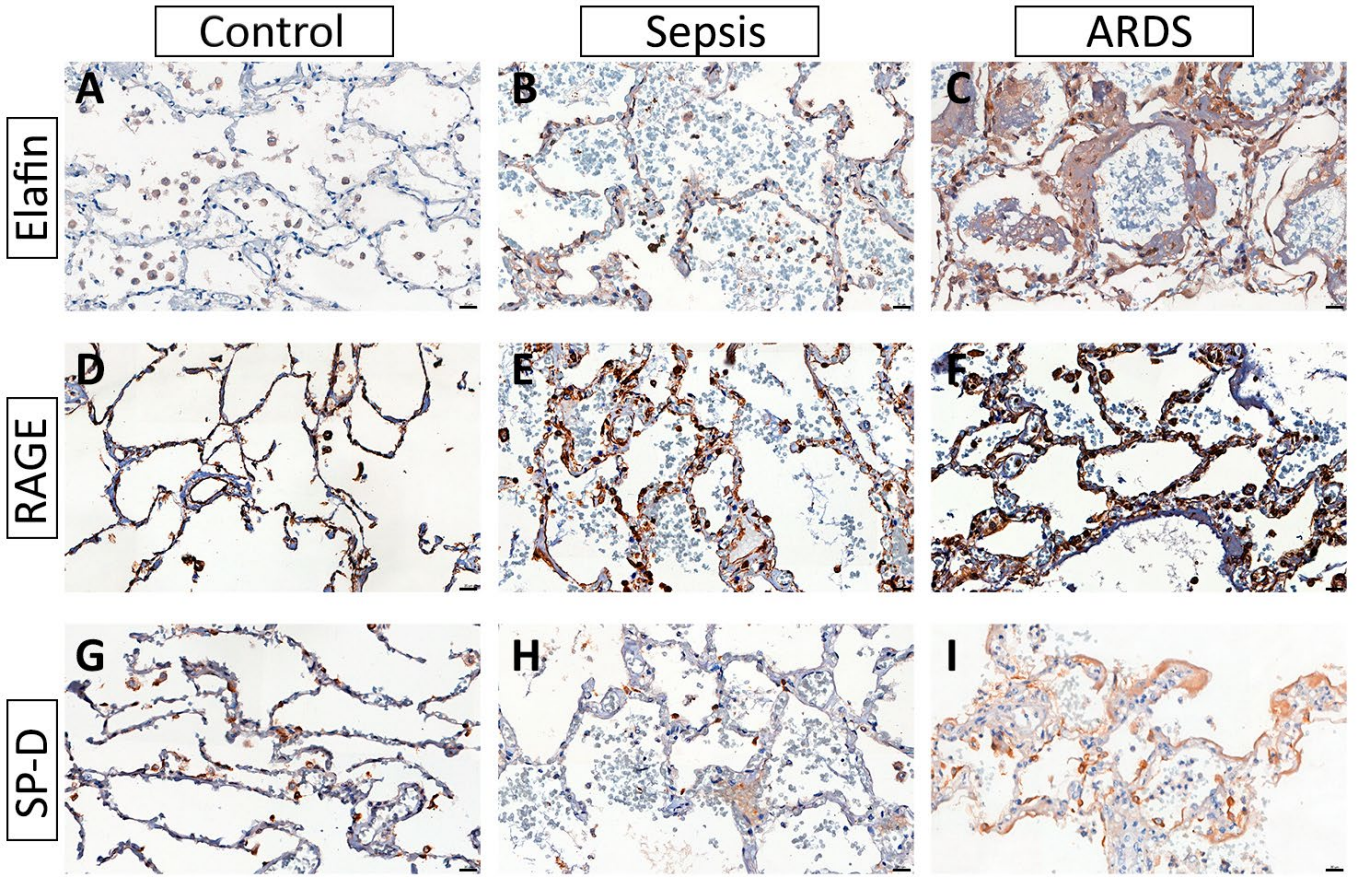
Representative Immunostained Photomicrographs of Control, Sepsis and ARDS lung tissue. Elafin: **A** – Control group: staining almost negative, except for some mildly positive macrophages; **B** – Sepsis Group: mild positive staining, mainly in lymphomononuclear cells; and **C**- ARDS group: Intense positive staining in epithelial and inflammatory cells. RAGE: **D**- Control group: intense positive staining of the alveolar cells and macrophages. **E** – Sepsis Group: intense positive staining of the alveolar cells and inflammatory cells. **F** – ARDS group: intense positive staining of the alveolar cells and inflammatory cells. SP-D: **G** – Control Group: Positive staining in type II pneumocytes. **H** – Sepsis Group: Positive staining in type II pneumocytes and mild positive staining in macrophages. **I** – ARDS Group: Positive staining of type II pneumocytes. Mild positive staining in macrophages and hyaline membrane. Scale Bar 40x = 20 μm

Figure 2 shows the photomicrographs of the elafin, RAGE and SP-D immunostaining. In normal conditions, elafin immunostaining is almost negative, except for some mildly positive macrophages (Fig. 2A). The sepsis group shows mild positive staining, mainly in lymphomononuclear cells (Fig. 2B) and the ARDS group shows intense positive staining in epithelial and inflammatory cells (Fig. 2C). In all groups, RAGE is highly expressed in the alveolar septa (Fig. 2D-F). In addition, the inflammatory cells display intense RAGE-positive staining in sepsis (Fig. 2E) and ARDS (Fig. 2F) groups. All groups show an SP-D positive staining of type II pneumocytes (Fig. 2G-I). Macrophages may present a mild SP-D positive staining because they can phagocyte surfactant proteins. The hyaline membrane is also SP-D-positive in the ARDS group (Fig. 2I).

The expression of all markers was heterogeneous within each case. Elafin showed a mean CV of 93% in the control group, 85% in the sepsis group, and 87% in the ARDS group. RAGE expression presented a mean CV of 32% in the control group, 36% in the sepsis group, and 35% in the ARDS group. SP-D expression CV was the only one with statistical differences among the groups. SP-D mean CV of the control (61%) group was significantly lower than the groups sepsis (87%; *p* < 0.0001) and ARDS (92%; *p* < 0.0001).

**Fig. 3:**
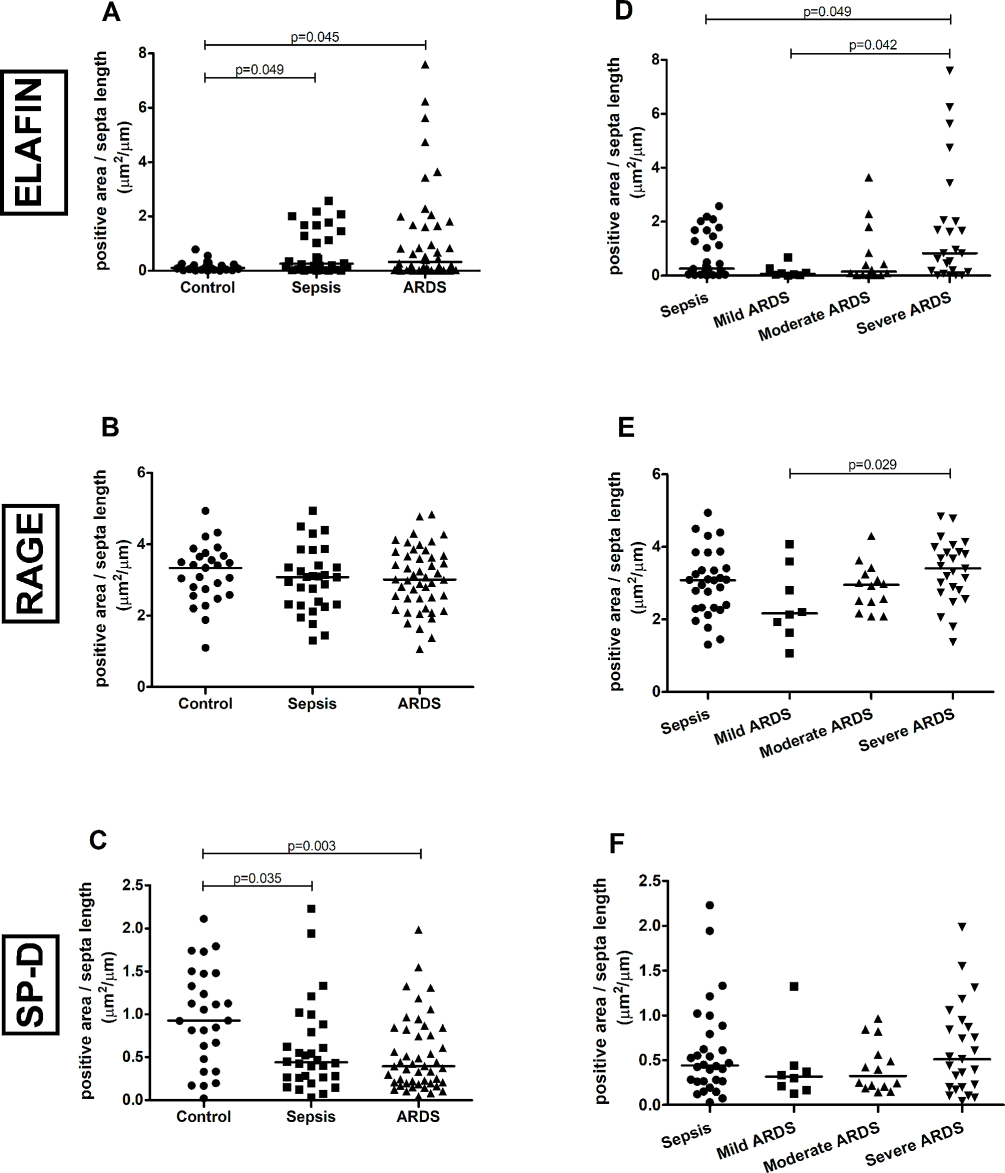
Graphical representation of protein expression of Elafin, RAGE and SP-D assessed by immunohistochemistry. **A**, **B**, and **C**: comparison among the control (*n* = 27), sepsis (*n* = 30) and ARDS groups (*n* = 47). **D**, **E**, and **F**: comparison among the sepsis (*n* = 30), mild ARDS (*n* = 8), moderate ARDS (*n* = 14), and severe ARDS (*n* = 25)

The comparison among the control, sepsis and ARDS groups showed an increase in the elafin expression in the ARDS (*p* = 0.045) and sepsis (*p* = 0.049) groups compared to the control group (Fig. 3A). We observed no difference in the RAGE expression among the groups (Fig. 3B). In addition, we observed a decrease in the SP-D expression in the ARDS (*p* = 0.003) and sepsis (*p* = 0.035) groups compared to the control group (Fig. 3C).

We have divided the ARDS group according to its severity and compared these new subgroups to the sepsis group, and we observed an increased elafin expression in the severe ARDS compared to the mild ARDS (*p* = 0.042) and sepsis cases (*p* = 0.049) (Fig. 3D). We also observed increased expression of RAGE in the severe ARDS compared to the mild ARDS (*p* = 0.029) (Fig. 3E). We observed no difference in the SP-D expression among these groups (Fig. 3F). Within the ARDS group, we only observed a tendency towards the increase of elafin in the pulmonary ARDS compared to the extrapulmonary ARDS (*p* = 0.05).

### Correlation between epithelial injury markers, histological evaluation, and clinical data

**Fig. 4:**
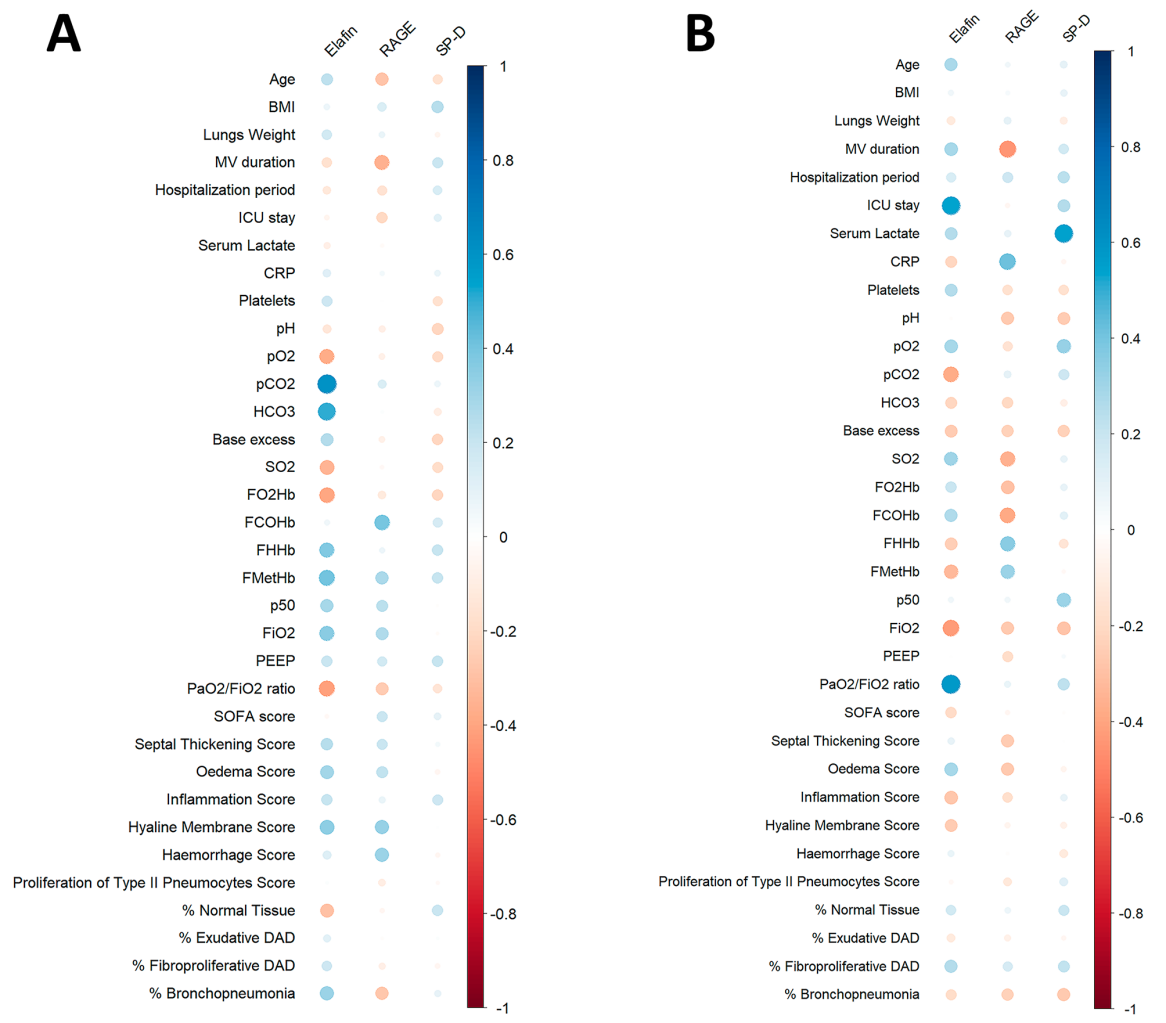
Heatmap of correlations between the epithelial injury markers and clinical variables and laboratory exams. **A** – ARDS group (*n* = 47). **B** – Sepsis group (*n* = 30). The correlation coefficients are color-coded from deep red (− 1) to deep blue (1)

**Fig. 5:**
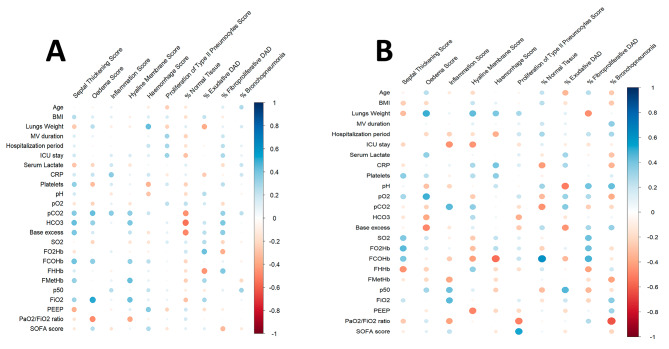
Heatmap of correlations between semi-quantitative histological assessment and clinical variables and laboratory exams. **A** – ARDS group (*n* = 47). **B** – Sepsis group (*n* = 30). The correlation coefficients are color-coded from deep red (− 1) to deep blue (1)

The correlations between the epithelial injury markers and histological evaluation, and clinical data are shown in Fig. 4. The correlations between the semi-quantitative histological assessment and clinical data are shown in Fig. 5. The specific correlation coefficients and *p*-values of the statistically significant correlations are shown in Tables 4 and 5.

**Table 4:** Specific correlation coefficient and *p*-values of the significant correlations between epithelial injury markers, histological evaluation, and clinical data in ARDS cases

Correlation between		Correlation Coefficient (r)	*p*-value
Elafin	pO_2_	-0.376	0.013
	pCO_2_	0.607	< 0.0001
	HCO_3_	0.509	< 0.0001
	SO_2_	-0,344	0.024
	FO_2_Hb	-0.398	0.027
	FMetHb	0.402	0.028
	FiO_2_	0.360	0.014
	PaO_2_/FiO_2_ ratio	-0.414	0.004
	% Normal lung tissue	-0.295	0.046
	% Bronchopneumonia	0.320	0.03
	Hyaline membrane score	0.342	0.02
	Oedema score	-0.298	0.044
RAGE	FCO_2_Hb	0.395	0.025
	PaO_2_/FiO_2_ ratio	-0.298	0.045
	MV duration	-0.354	0.015
	Haemorrhage score	0.318	0.029
	Hyaline membrane score	0.318	0.029
% Normal lung tissue	pCO2	-0.457	0.002
	HCO3	-0.544	< 0.0001
	Base excess	-0.483	0.001
% Exudative DAD	FO2Hb	0.413	0.019
	FHHb	-0.447	0.010
% Fibroproliferative DAD	pCO_2_	0.324	0.032
	HCO_3_	0.382	0.010
	Base excess	0.346	0.021
	FHHb	0.378	0.033
	FO_2_Hb	-0.362	0.042
	SOFA score	-0.326	0.034
Septal thickening score	Platelets	0.330	0.025
	pCO_2_	0.362	0.016
	HCO_3_	0.428	0.004
	Base excess	0.383	0.010
	FCO_2_Hb	0.403	0.022
Oedema score	Platelets	-0.310	0.036
	pCO_2_	0.420	0.004
	HCO_3_	0.321	0.034
	FiO_2_	0.513	< 0.0001
	PaO_2_/FiO_2_ ratio	-0.474	0.001
Inflammation score	pCO_2_	0.350	0.02
Haemorrhage score	Lungs’ weight	0.429	0.003
	Platelets	-0.346	0.018
Hyaline membrane score	pCO_2_	0.347	0.021
	HCO3	0.384	0.010
	FMetHb	0.425	0.017
	FiO_2_	0.419	0.003
	PaO_2_/FiO_2_ ratio	-0.394	0.006

**Table 5:** Specific correlation coefficient and *p*-values of the significant correlations between epithelial injury markers, histological evaluation, and clinical data in sepsis cases

Correlation between		Correlation Coefficient (r)	*p*-value
Elafin	ICU stay	0.533	0.015
RAGE	C-reactive protein	0.418	0.047
	MV duration	-0.422	0.014
SP-D	Serum lactate	0.540	0.008
% Normal lung tissue	C-reactive protein	-0.429	0.041
	FCO_2_Hb	0.603	0.010
% Exudative DAD	Arterial blood pH	-0.512	0.025
% Fibroproliferative DAD	Lungs’ weight	-0.468	0.009
% Bronchopneumonia	PaO_2_/FiO_2_ ratio	-0.628	0.012
Oedema score	Lungs’ weight	0.434	0.016
	pO_2_	0.484	0.036
	Base excess	-0.482	0.037
Haemorrhage score	Lungs’ weight	0.381	0.038
	FCOHb	-0.555	0.021

## Discussion

Amongst our main results, we observed that all features of DAD were milder in septic group compared to ARDS group. We also observed that the protein expression of elafin is increased and SP-D is decreased in the sepsis and ARDS groups. The severe ARDS showed higher expression of elafin and RAGE in the lung tissue, and both correlated with several blood gas parameters, including a negative correlation with the PaO_2_/FiO_2_ ratio in the ARDS group. They also correlate with the percentage of bronchopneumonia and hyaline membrane score. In addition, RAGE expression was also negatively correlated with MV duration in both ARDS and septic groups. In septic patients, RAGE correlated with C-reactive protein, elafin was positively correlated with ICU stay, and SP-D was positively correlated with serum lactate.

DAD can be induced by a cascade of pathological events that culminate in damage to the alveolar-capillary barriers and to pulmonary homeostasis. Although considered the pathological hallmark of ARDS, DAD is a non-specific lung reaction to several conditions that may overlap, including sepsis, acute interstitial pneumonia, and trauma [15]. The injury of epithelial and endothelial cells can result in the disruption of the alveolar epithelial–capillary barrier that enhances the alveolar-capillary permeability, thereby allowing the leakage of serum proteins into air spaces while also facilitating the escape of proteins from the alveolar space into the bloodstream [16, 17].

All sepsis and ARDS cases included in this study showed at least some degree of DAD. However, some features of DAD were more intense in the ARDS group, such as increased lung weight, inflammation, hyaline membrane formation, septal thickening, and alveolar haemorrhage. The severity of lung epithelial injury in ARDS is an important determinant of patient survival [18]. Injured pneumocytes lose their tight barrier and polarity which decreases their ability to efficiently reabsorb fluid, exacerbating the pulmonary oedema formation. Therefore, measurement of impaired alveolar fluid clearance has been used to identify lung epithelial injury [18]. More recently, the assessment of specific markers in plasma, such as SP-D and RAGE has been suggested as a useful tool to assess lung epithelial injury [19].

We did not observe a difference in the RAGE expression amongst the group control, sepsis and ARDS. RAGE is constitutively highly expressed in the lungs, mainly produced by pneumocytes type I [20], and the expression by pneumocyte type II has also been reported [21]. RAGE is also expressed by a variety of immune cells, such as macrophages, dendritic cells and lymphocytes [20]. Even with the evident damage of the lung epithelial cells in the ARDS group, we did not observe a difference of RAGE tissue expression among the groups, probably due to the presence of numerous RAGE-expressing inflammatory cells in the ARDS lung tissue. Similarly, Wittkowski et al. [22] did not observe a significant RAGE protein expression difference in lung samples of ARDS and control patients.

Soluble RAGE (sRAGE), the cleaved form of the receptor, measured in plasma or BALF has been thought to be released in the lung due to alveolar epithelial and endothelial injury or alternatively, it may occur as part of a pulmonary inflammatory response. Regardless of the initiating stimulus, the translocation of sRAGE into the systemic circulation may be enhanced by increased alveolar-capillary permeability [23]. Thus, sRAGE level is proposed as a biomarker of type I alveolar cell injury and alveolar fluid clearance.

Briot et al. [24] showed that the alveolar fluid clearance rate was inversely correlated with levels of sRAGE assessed in the alveolar fluid from human lungs declined for transplantation. Mrozek et al. [25] found that plasma sRAGE was associated with a non-focal ARDS, that is suggestive of inflammatory oedema and impaired alveolar fluid clearance. Furthermore, plasma levels of sRAGE were correlated with ARDS mortality [26] and with the clinical severity of ARDS [27], decreasing over time, suggesting the resolution of alveolar epithelial injury [28]. Accordingly, we also observed an association between ARDS severity and RAGE tissue expression, demonstrated by the negative correlation between RAGE expression and PaO_2_/FiO_2_ ratio, and the significant increase of RAGE expression in the severe ARDS group compared to mild ARDS. In addition, we also observed a negative correlation of RAGE expression with MV duration in ARDS and sepsis groups.

Increased expression of RAGE in several other inflammatory diseases has been reported [20], including sepsis. It has been shown that plasma sRAGE levels increase during sepsis progression and severity [29] and that these levels are even higher in non-survivors [30]. Ware et al. [31] showed that abnormal levels of five plasma markers (SP-D, RAGE, IL-8, IL-6 and CC-16) provided valid discrimination for diagnosis of ARDS in patients with sepsis.

SP-D expression was decreased in the sepsis and ARDS groups compared to the control group. Similarly, Cheng et al. [32] observed that SP-D levels were lower in ARDS patients with worse oxygenation and in patients who did not survive. Greene et al. [33] found that just after ARDS onset, BALF SP-D concentration was significantly lower in patients who died and that it was correlated with the PaO_2_/FiO_2_ ratio. Increased leakage of pulmonary epithelium during lung injury may reduce the levels of surfactant proteins in BALF, with or without increment in its synthesis [34]. In accordance, early in the course of ARDS increased plasma levels of SP-D are associated with a worse clinical outcome and these levels are attenuated by protective lung mechanical ventilation with lower tidal volumes [35].

In the lungs, elafin is produced by epithelial and inflammatory cells that potently inhibit the neutrophil-derived elastase, protecting the lung tissue from the harmful effects of proteases [36]. We observed increased tissue expression of elafin in the sepsis and ARDS group. In the ARDS group, we also observed a negative correlation with the PaO_2_/FiO_2_ ratio and even more elevated expression of elafin in the severe ARDS group. Our data indicate that the worse the hypoxemia, the more elafin is produced locally in the lung tissue.

Sallenave et al. [37] showed that the concentrations of elafin were increased in BALF of ARDS patients, however, there was no significant difference between patients with ARDS and those at risk but without ARDS. Other studies observed that elafin levels were increased in the serum of patients at the onset of ARDS [7] but decreased along with the progress of ARDS [7, 38, 39]. In addition, Wang et al. [40] showed that serum elafin levels were even lower in non-survivors ARDS patients compared to survivor patients. In our study, we did not observe any correlation between the elafin protein expression and variables that may suggest any variation along the temporal course of ARDS. Notably, Kerrin et al. [7] demonstrated that the BALF elafin concentrations fall over the course of ARDS was a result of its proteolytic degradation, while Wang et al. [38] hypothesized that elafin decline in serum was due to its binding to the extracellular matrix. Both assumptions suggest that it may be hard to accurately assess the levels of elafin in BALF or serum.

According to previous studies approximately 50% of cases with clinical criteria for ARDS have DAD on lung histology. DAD seems to be more frequent in more severe cases of ARDS and patients with DAD have higher mortality [6, 8, 41]. Considering that DAD and loss of epithelial-capillary barrier integrity are factors that favor the translocation of proteins from the lung into the bloodstream, these markers of epithelial injury may not be good predictors for cases that have clinical criteria for ARDS, but not have significant DAD. However, these markers can be good discriminators of a subpopulation of patients with a more uniform histological diagnosis and a worse prognosis.

Our study has some limitations due to the difficulty of addressing the multifactorial nature of ARDS and the multiple conditions that may overlap in critical patients. Our controls were not matched by gender, age, or MV duration and settings. In addition, the limited number of subjects does not allow us to address all possible confounding factors. Since it is a retrospective study and only tissue was available for analysis, we could not assess how the lung injury would reflect on BALF or plasma concentrations of elafin, RAGE, and SP-D. Despite these limitations, our findings contribute to the existing body of knowledge on ARDS.

In summary, lung tissue expression of elafin and RAGE, but not SP-D, is associated with ARDS severity, but does not discriminate sepsis patients from ARDS patients.

### Electronic supplementary material

Below is the link to the electronic supplementary material.

Supplementary Material 1. Table S1. Clinical data from sepsis and ARDS group. Figure S1. (A) Simple correspondence analysis of the septal thickening score biplot. (B) Heatmap of the adjusted standardized residuals from the chi-square test. Figure S2. (A) Simple correspondence analysis of the oedema score biplot. (B) Heatmap of the adjusted standardized residuals from the chi-square test. Figure S3. (A) Simple correspondence analysis of the inflammation score biplot. (B) Heatmap of the adjusted standardized residuals from the chi-square test. Figure S4. (A) Simple correspondence analysis of the hyaline membrane score biplot. (B) Heatmap of the adjusted standardized residuals from the chi-square test. Figure S5. (A) Simple correspondence analysis of the alveolar haemorrhage score biplot. (B) Heatmap of the adjusted standardized residuals from the chi-square test. Figure S6. (A) Simple correspondence analysis of the proliferation of type II pneumocytes score biplot. (B) Heatmap of the adjusted standardized residuals from the chi-square test. Figure S7. (A) Simple correspondence analysis of the inflammation pattern biplot. (B) Heatmap of the adjusted standardized residuals from the chi-square test. Figure S8. Semi-quantitative histological analysis of the Pulmonary ARDS and Extrapulmonary ARDS groups.

## Data Availability

The datasets supporting the conclusions of this article are included within the article and its additional file. Further information is available from the corresponding author upon reasonable request.
