# Supplementary material for: Lung tissue expression of epithelial injury markers is associated with acute lung injury severity but does not discriminate sepsis from ARDS
Source: Respir Res. 2024 Mar 18;25:129. doi: 10.1186/s12931-024-02761-x (PMC10949726; doi:10.1186/s12931-024-02761-x)
Supplement: Supplementary file 1 — Supplementary Material 1. Table S1. Clinical data from sepsis and ARDS group. Figure S1. (A) Simple correspondence analysis of the septal thickening score biplot. (B) Heatmap of the adjusted standardized residuals from the chi-square test. Figure S2. (A) Simple correspondence analysis of the oedema score biplot. (B) Heatmap of the adjusted standardized residuals from the chi-square test. Figure S3. (A) Simple correspondence analysis of the inflammation score biplot. (B) Heatmap of the adjusted standardized residuals from the chi-square test. Figure S4. (A) Simple correspondence analysis of the hyaline membrane score biplot. (B) Heatmap of the adjusted standardized residuals from the chi-square test. Figure S5. (A) Simple correspondence analysis of the alveolar haemorrhage score biplot. (B) Heatmap of the adjusted standardized residuals from the chi-square test. Figure S6. (A) Simple correspondence analysis of the proliferation of type II pneumocytes score biplot. (B) Heatmap of the adjusted standardized residuals from the chi-square test. Figure S7. (A) Simple correspondence analysis of the inflammation pattern biplot. (B) Heatmap of the adjusted standardized residuals from the chi-square test. Figure S8. Semi-quantitative histological analysis of the Pulmonary ARDS and Extrapulmonary ARDS groups. [file 12931_2024_2761_MOESM1_ESM.docx]

**Supplementary Material**

**Table S1.** Clinical data from sepsis and ARDS group.

|  | **Sepsis** | | **ARDS** | | **p-value** |
| --- | --- | --- | --- | --- | --- |
|  | **n** | **Median (IQR)** | **n** | **Median (IQR)** |  |
| **Arterial blood gas analysis** |  |  |  |  |  |
| pH | 19 | 7.08 (0.29) | 47 | 7.15 (0.25) | 0.172 |
| pO_2_ (mmHg) | 19 | 86.9 (46.1) | 47 | 81 (38.1) | 0.118 |
| pCO_2_ (mmHg) | 19 | 32.5 (14.3) | 47 | 37.7 (25.1) | **0.049** |
| HCO_3_ (mmol/L) | 19 | 10.5 (7.5) | 47 | 15.05 (9.6) | **0.005** |
| Base Excess (mmol/L) | 19 | -19.1 (8.7) | 47 | -12.5 (12.6) | **0.004** |
| SO_2_ (%) | 19 | 89.6 (10.5) | 47 | 88.5 (14.8) | 0.385 |
| FO_2_Hb (%) | 17 | 89.4 (13.8) | 32 | 86.1 (15.5) | 0.323 |
| FCO_2_Hb (%) | 17 | 1.4 (0.8) | 32 | 0.9 (1) | 0.135 |
| FHHb (%) | 17 | 9 (14) | 32 | 11.5 (15) | 0.248 |
| FMetHb (%) | 17 | 0.7 (0.4) | 32 | 0.8 (0.6) | 0.386 |
| p50 (mmHg) | 17 | 38 (10) | 32 | 32 (8) | **0.009** |
| **Serum Lactate (mg/dL)** | 30 | 125 (120) | 46 | 45.5 (82) | **0.003** |
| **CRP (mg/L)** | 23 | 146 (175.9) | 28 | 234.4 (212.3) | **0.042** |
| **Blood cell counts** |  |  |  |  |  |
| Erythrocytes (million/mm^3^) | 24 | 2.9 (0.96) | 44 | 2.9 (0.64) | 0.505 |
| Haemoglobin (g/dL) | 25 | 8 (1.9) | 44 | 8.6 (2.1) | 0.685 |
| Haematocrit (%) | 25 | 26.4 (4.8) | 44 | 26.8 (6.9) | 0.631 |
| Leucocytes (1.000/mm^3^) | 25 | 18.78 (21.33) | 45 | 11.14 (21.33) | 0.106 |
| Neutrophils (1.000/mm^3^) | 24 | 15.8 (21.7) | 40 | 12.75 (19) | 0.325 |
| Segmented Neutrophils (1.000/mm^3^) | 18 | 18.15 (20) | 28 | 15.6 (16.8) | 0.323 |
| Metamyelocyte (1.000/mm^3^) | 12 | 0.5 (1) | 23 | 0.12 (0) | **0.028** |
| Myelocyte (1.000/mm^3^) | 6 | 0.6 (0) | 19 | 0 (0) | **0.003** |
| Promyelocyte (1.000/mm^3^) | 2 | 0.5 | 14 | 0 (0) | 0.067 |
| Lymphocytes (1.000/mm^3^) | 24 | 1.45 (3.4) | 40 | 1.15 (1.1) | 0.122 |
| Platelets (1.000/mm^3^) | 25 | 112 (120) | 46 | 79 (123) | 0.028 |


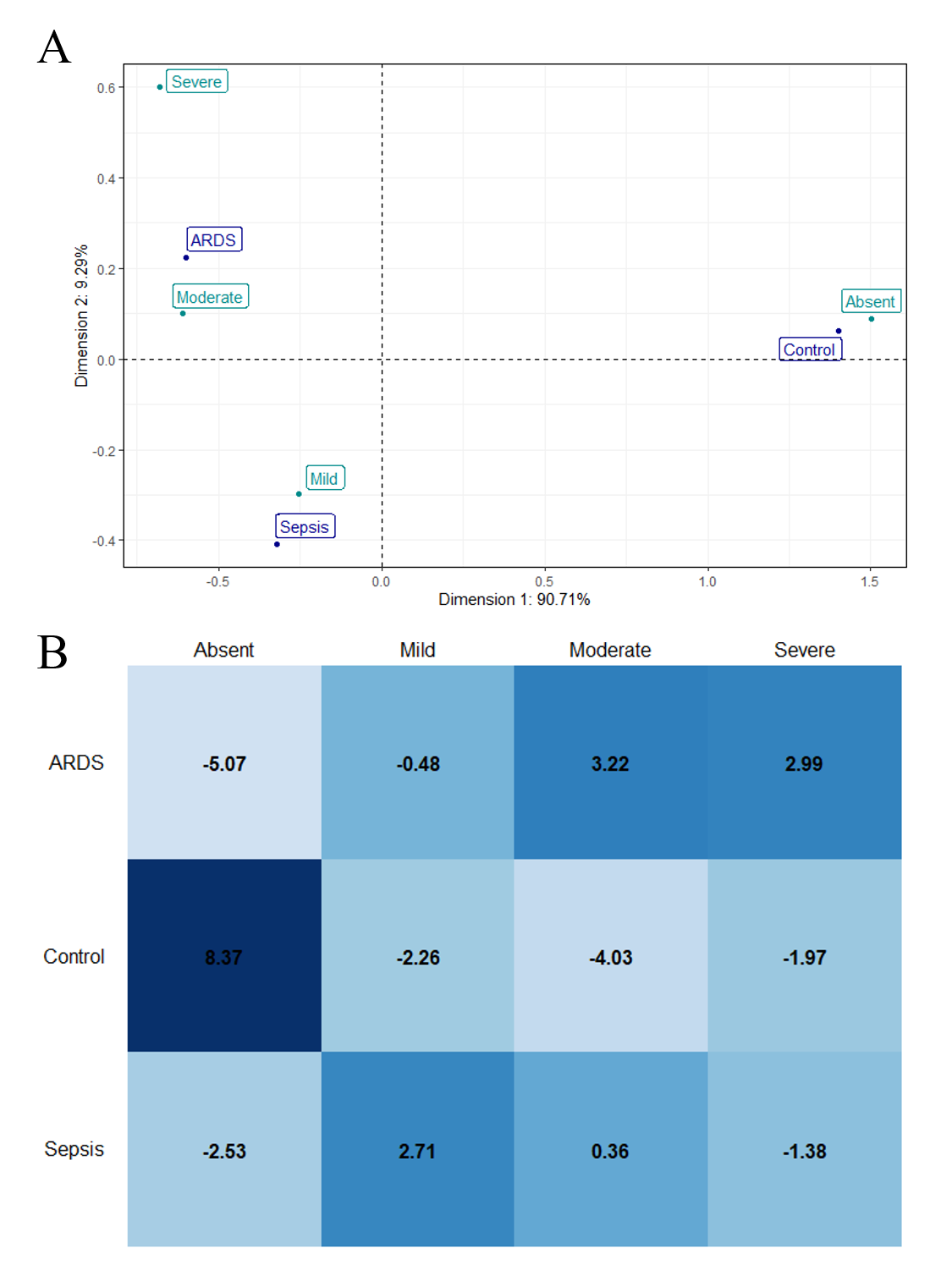


**Figure S1.** (A) Simple correspondence analysis of the septal thickening score biplot. (B) Heatmap of the adjusted standardized residuals from the chi-square test. Chi-Squared =80.634; p-value < 0.0001.


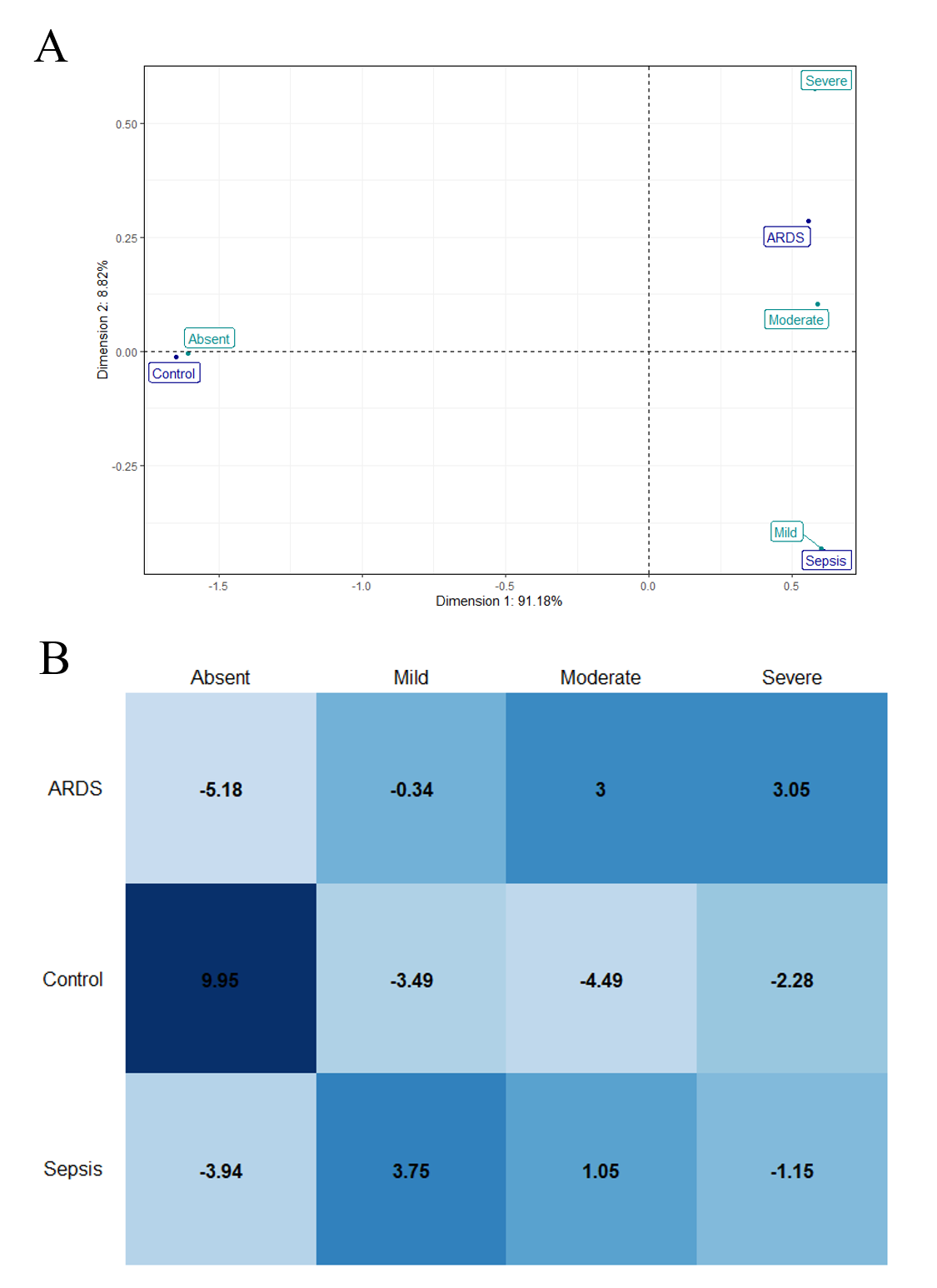


**Figure S2.** (A) Simple correspondence analysis of the oedema score biplot. (B) Heatmap of the adjusted standardized residuals from the chi-square test. Chi-Squared =108.607; p-value < 0.0001.


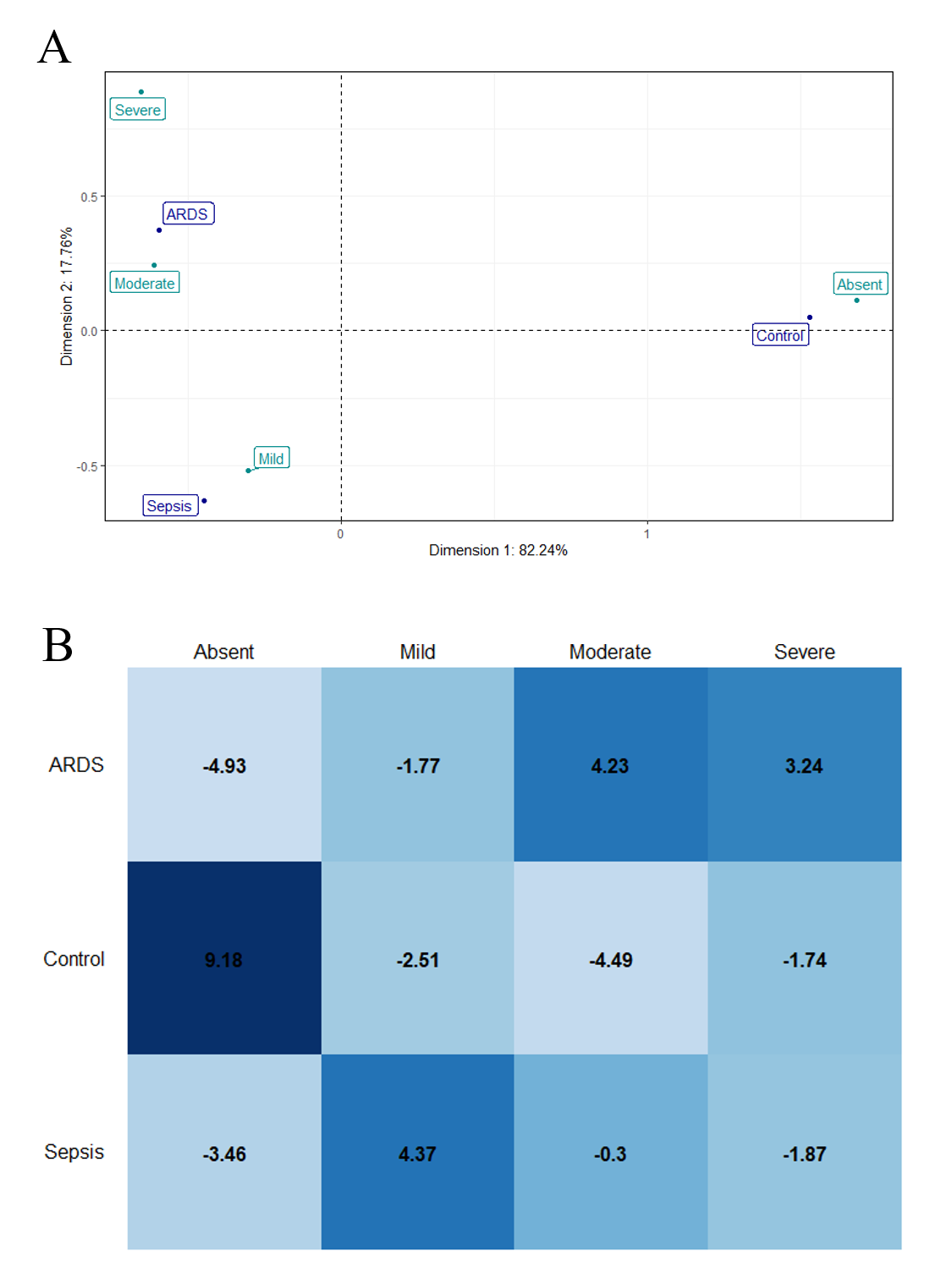


**Figure S3.** (A) Simple correspondence analysis of the inflammation score biplot. (B) Heatmap of the adjusted standardized residuals from the chi-square test. Chi-Squared =104.343; p-value < 0.0001.


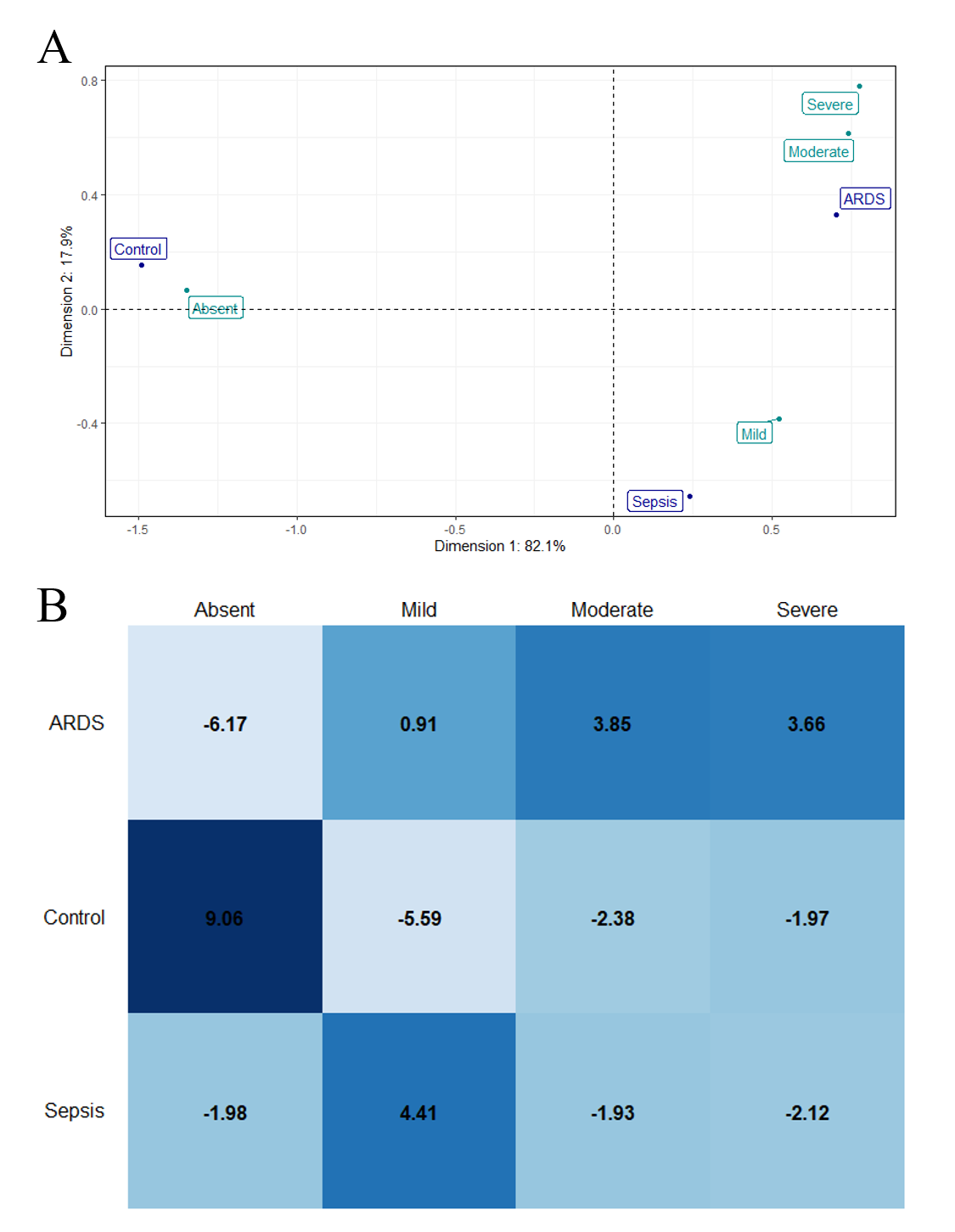


**Figure S4.** (A) Simple correspondence analysis of the hyaline membrane score biplot. (B) Heatmap of the adjusted standardized residuals from the chi-square test. Chi-Squared =103.698; p-value < 0.0001.


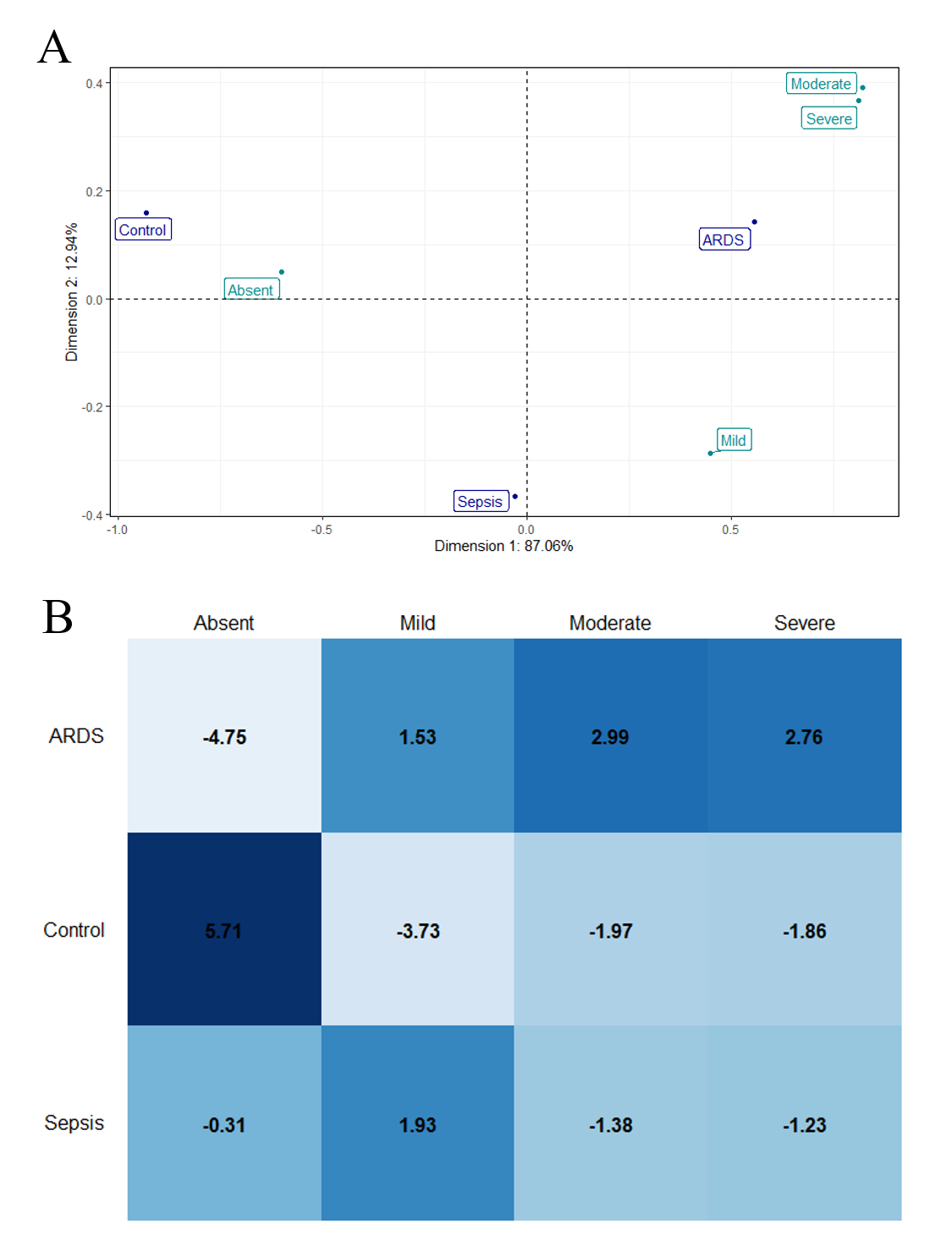


**Figure S5.** (A) Simple correspondence analysis of the alveolar haemorrhage score biplot. (B) Heatmap of the adjusted standardized residuals from the chi-square test. Chi-Squared =43.611; p-value < 0.0001.


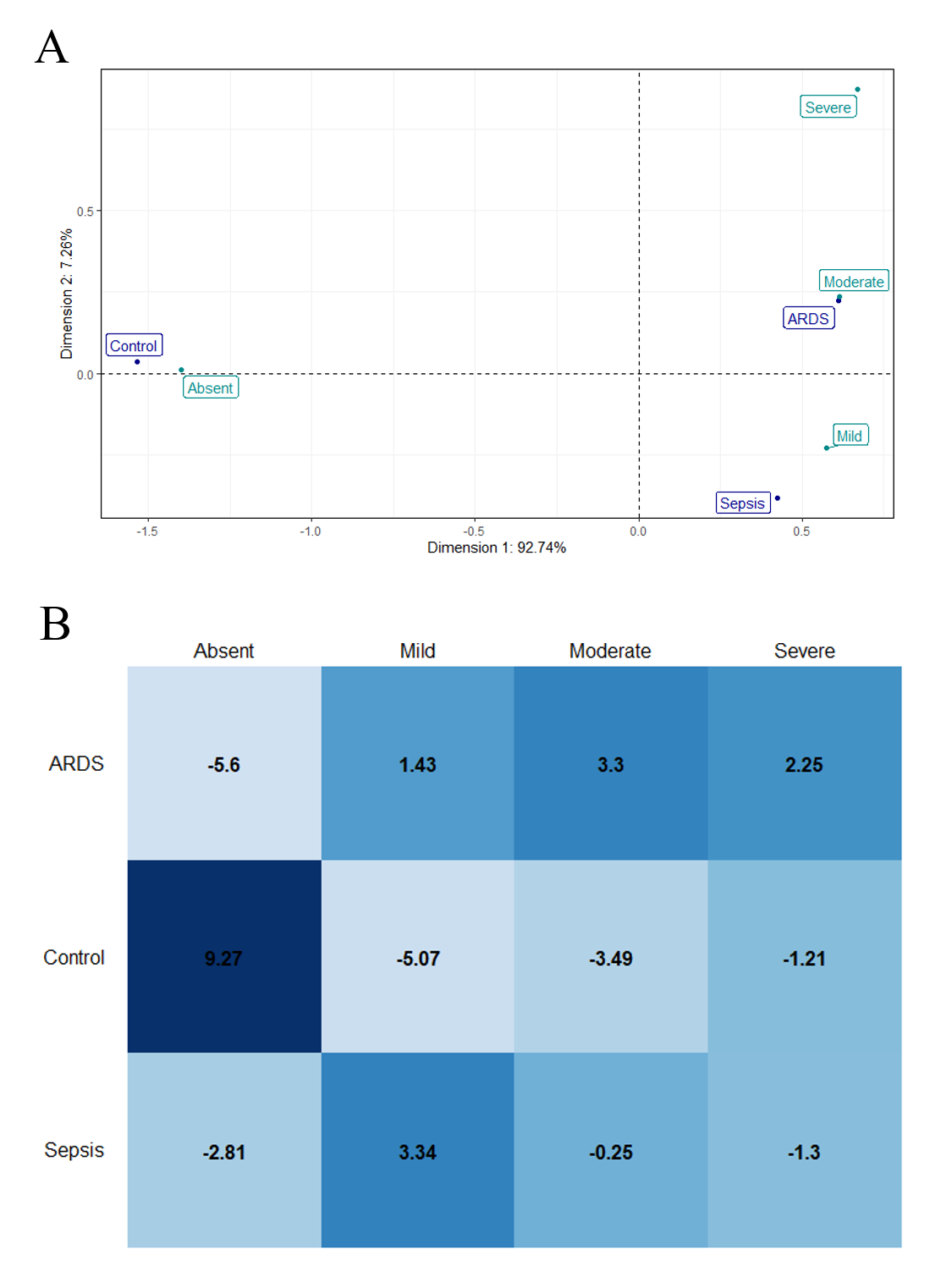


**Figure S6.** (A) Simple correspondence analysis of the proliferation of type II pneumocytes score biplot. (B) Heatmap of the adjusted standardized residuals from the chi-square test. Chi-Squared =93.237; p-value < 0.0001.


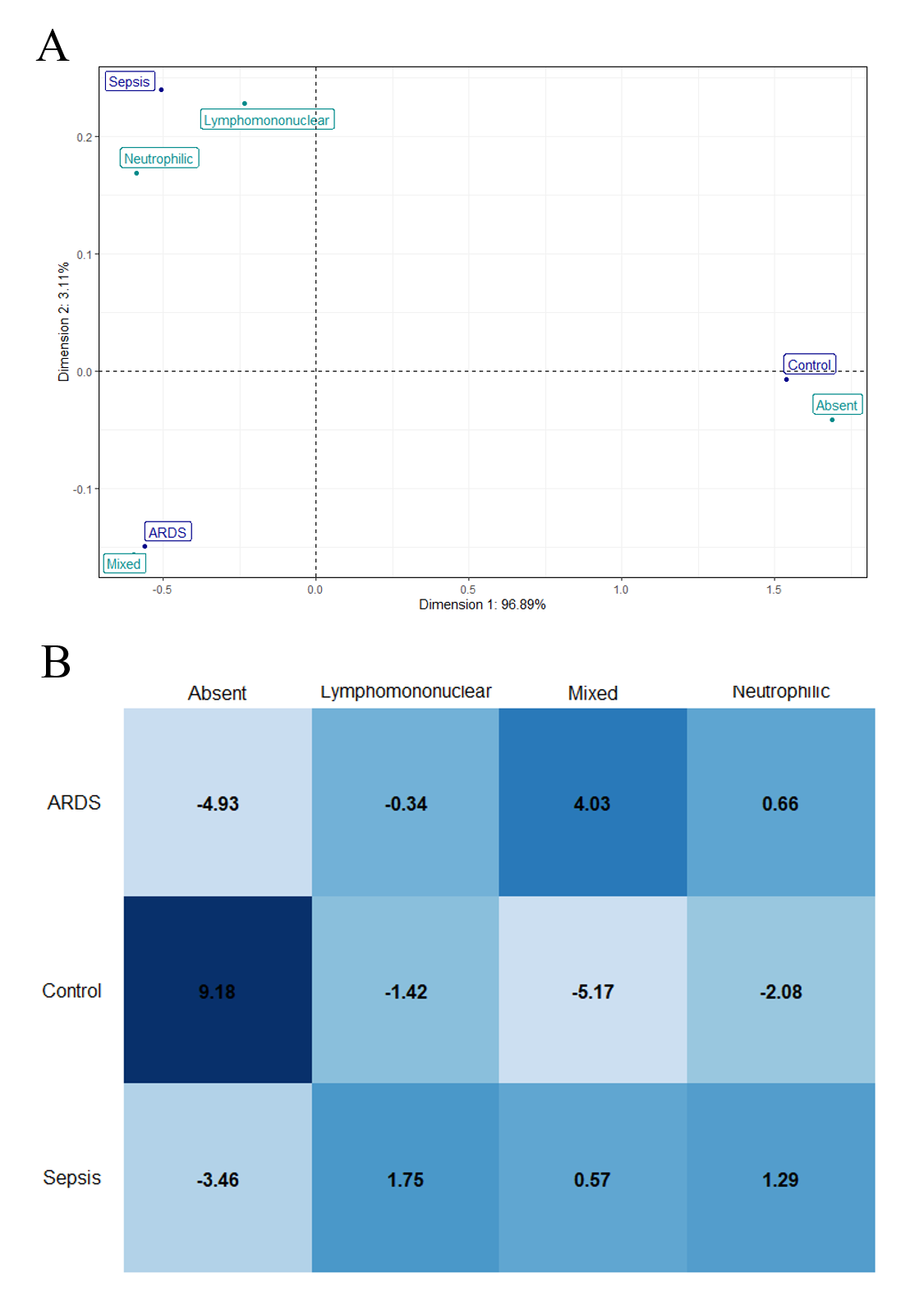


**Figure S7.** (A) Simple correspondence analysis of the inflammation pattern biplot. (B) Heatmap of the adjusted standardized residuals from the chi-square test. Chi-Squared =89.218; p-value < 0.0001.


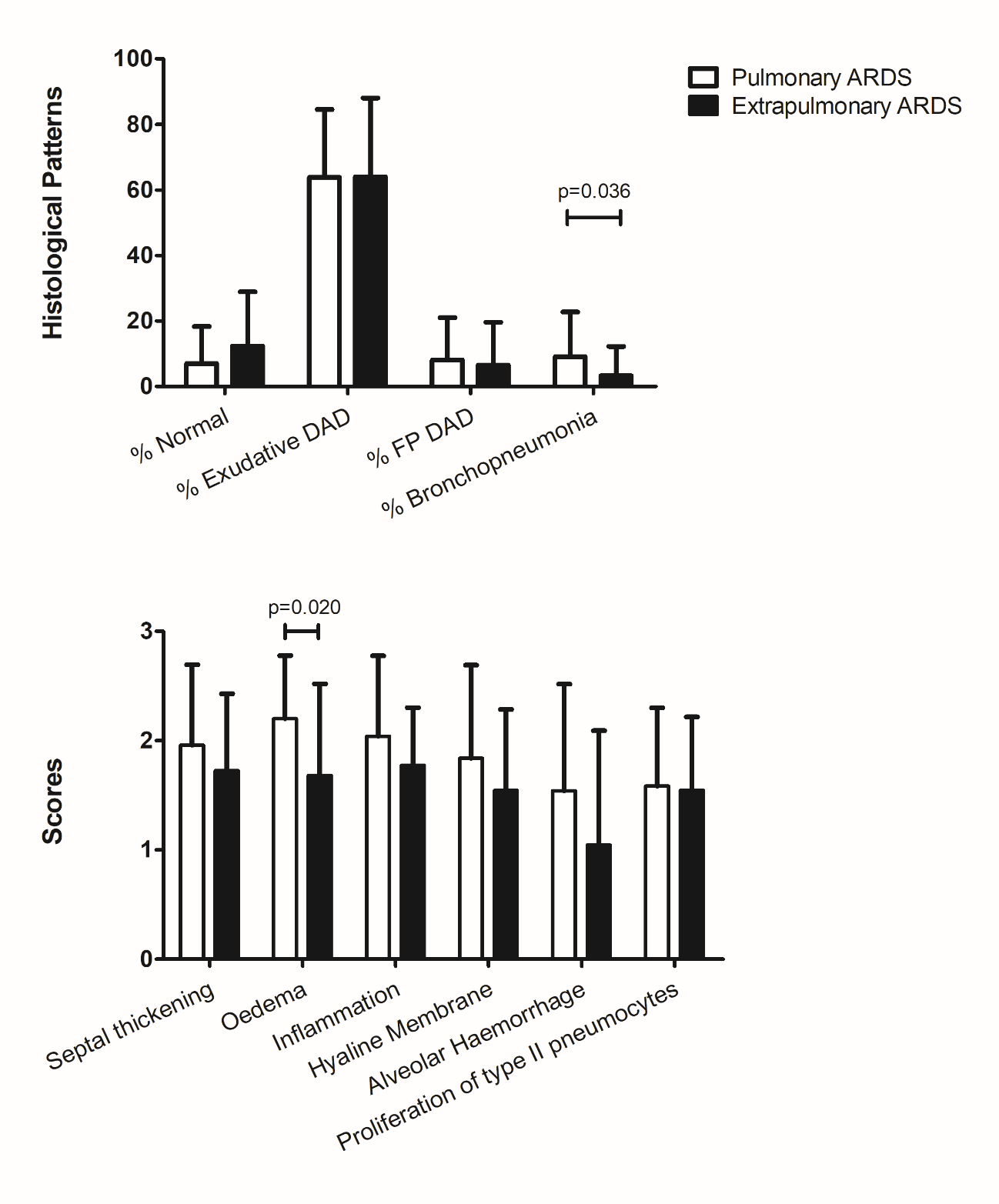


**Figure S8.** Semi-quantitative histological analysis of the Pulmonary ARDS and Extrapulmonary ARDS groups.
